# Factors Associated with Healthcare Services Utilization and Pharmacological Treatment in Individuals with Diabetes Diagnosis: Lessons from a Nationwide Program for Diabetes Mellitus Detection in Brazil

**DOI:** 10.5402/2011/342071

**Published:** 2011-10-31

**Authors:** Maria Isabel Fischer, Cristiana Maria Toscano, Sotero Serrate Mengue

**Affiliations:** Programa de Pós Graduação em Epidemiologia, Faculdade de Medicina, Universidade Federal do Rio Grande do Sul, Avenida Anita Garibaldi, 1921/607, 90480-201 Porto Alegre, RS, Brazil

## Abstract

The Brazilian Nationwide Population Screening Program for Diabetes, conducted in 2001, diagnosed 346,168 new cases. Although unexpected, approximately 65,000 previously diabetic individuals participated. We describe their characteristics compared to new cases, based on data obtained by a follow-up study of a subsample of 4991 positively screened from a representative sample of 90,106 individuals. Two groups were analyzed regarding factors associated with adherence to treatment, healthcare services utilization, and compliance to pharmacological treatment: 497 with newly diagnosed diabetes and 257 individuals with previous diabetes diagnosis who were not under treatment at the screening program. For this group, healthcare service utilization was lower when compared with the new cases (OR = 0.06; 95% CI: 0.03–0.12). Diabetes status (OR = 0.23; 95% CI: 0.14–0.37), a healthy behavior score (OR = 0.53, 95% CI: 0.34–0.83), and glucose levels at screening (altered, OR = 5.01; 95% CI: 2.38–10.6 and likely and very likely DM OR = 11.2; 95% CI: 6.85–18.4) were independently associated with pharmacological treatment.

## 1. Introduction

Worldwide, the prevalence of diabetes mellitus (DM) continuous to rise, being an international health burden (1). The increase of individuals with DM is expected to happen mainly in developing countries due to the growth and aging of populations, urbanization, obesity, and sedentary behavior [1–3]. In Brazil a 69% increase in the number of people with diabetes for the year 2030 is estimated, compared to 2000 [4].

In 2001, the Brazilian Ministry of Health, with the collaboration of state and municipal health authorities and medical societies, implemented a National Campaign for the Reorganization of Diabetes Mellitus Care (CNDDM) [5]. 

The Brazilian Nationwide Population Screening Program for Diabetes (BNPSPD), was one of several components of the National Plan, being conducted through primary healthcare services during March 6th and April 7th, 2001 and have been previously described [5, 6]. Briefly, all Brazilian citizens aged 40 or older were invited to participate, and 22.069,905 capillary glucose tests were performed, of which 15.48% were considered positive (fasting capillary glucose ≥ 100 mg/dL or casual glucose ≥ 140 mg/dL). Screening was performed in a “campaign” style, in which the population was called for primary health care services in a short period of time to perform screening tests [7, 8]. 

This study was proposed considering the magnitude of the results obtained in the BNPSPD and the difficulties perceived and presented in other investigations [9–12] such as adherence to treatment and healthy behavior in chronic disease. The main objective of this study was to describe a group of individuals who were not investigated in the literature so far: those who were identified as having diabetes diagnosis during the BNPSPD have reported having this diagnosis prior to screening and, despite this diagnosis, they were not under medical treatment. We also aimed to identify factors associated to healthcare services utilization and compliance to pharmacological treatment in those individuals compared to newly diagnosed DM cases.

## 2. Methods

A detailed description of the follow-up study of individuals positively screened during the screening program is described in detail elsewhere [8, 11]. In summary, from a follow-up study of a representative stratified random sample of 90,106 a subsample of 4991 positively screened individuals was actively followed up through home interviews which were performed by trained professionals using a standardized questionnaire 15 to 19 months after screening. The questionnaire included 43 questions on demographics, BNPSPD participation, diagnostic confirmation, and orientation received and adopted to treat diabetes, laboratory tests, therapy and use of medications, and followup in healthcare services. 

**Figure 1:**
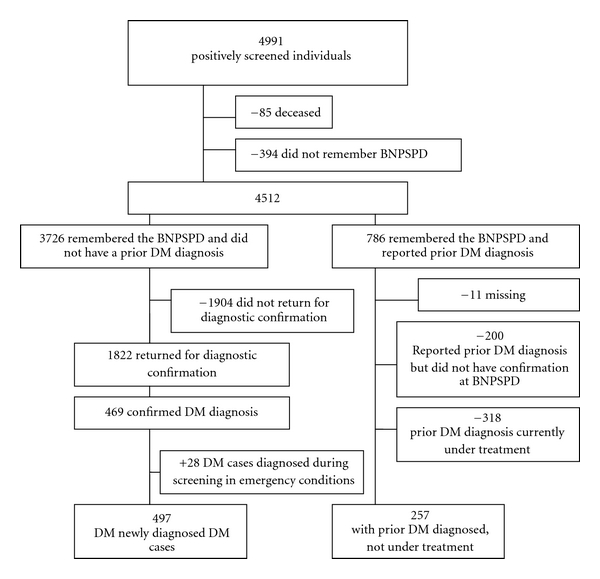
Sample of positively screened individuals during the BNPSPD, included in the follow-up study, Brazil, 2002. DM, diabetes mellitus; BNPSPD: Brazilian Nationwide Population Screening Program for Diabetes.

Among 4991 (Figure 1) participants that were interviewed and that had positive screening tests, 85 were deceased at the moment of the follow-up study and the 394 who did not remember participating in the CNDDM were excluded. Of the 4512 remaining individuals, 786 declared that they knew that they were diabetic at the screening, with 257 confirmed as diabetics that did not treat the condition. Among the 3726 participants that were alive, remembered CNDDM participation, and were not previously diabetic, 1822 were submitted to tests and 469 had their diabetes confirmed. An additional 28 individuals had their diabetes confirmed by a combination of medical assistance in emergency conditions and a high blood glucose level during the screening, for a total of 497 new cases detected (NCD, reference group). Therefore, two groups were formed for analysis: new cases (497) and previously diabetic individuals that were not under treatment (PDWIT, 257). This group was defined as those that declared not to be treating diabetes in the CNDDM and declared, at the active surveillance, that they knew they were diabetic before the screening. 

For the screening program, standardization and classification were defined for tests results according to which recommendations were made to individuals who participated varying from repeat test in 3 years to immediate consultation with physician (Table 1). For the purposes of this study, individuals presenting glucose blood tests at the level of likely (fasting or casual ≥ 200) or very likely (fasting or casual ≥ 270 mg/dL) diabetes at screening and those who had a confirmatory test were considered having diabetes. No additional biochemical assessments were done in patients, except for glucose testing. A third group of 318 previously diabetic individuals that had their diagnoses confirmed was excluded from this study because participants declared to be treating their diabetes before CNDDM, although in the form at screening they declared that they did not treat the condition (11). 

Gender, age, schooling, glucose blood test result in the program, adhesion to pharmacological and nonpharmacological treatment for diabetes, and health care services utilization were analyzed. Individuals who informed to be under medical followup were considered utilizing health care services. In order to quantify adherence to non-pharmacological recommendations, a numeric score (from 0–7) was developed in which one point was given to each healthy behavior referred by the individual: weight control, ingestion of food with low levels or no salt, preference to low-fat food, ingestion of fruits or vegetables at least twice a day, avoiding or not smoking, regular physical activity, and monitoring glycemic levels at least once every three years.

All data were collected on standardized forms, double entered into an electronic database, and analyzed using the survey data analysis function of STATA, version 8.2. Univariate analysis was performed considering the sampled primary healthcare units (PHUs) as the basic unit of the conglomerate (PSU, primary strata unit) while subnational regions were considered as the strata. Odds ratios and 95% confidence intervals were estimated for variables analyzed. All variables significantly associated with healthcare services utilization and compliance to pharmacological treatment in bivariate analysis (*P* ≤ 0.05) were included in the logistic regression model.

## 3. Results

A total of 497 individuals in the NCD group (control group) and 257 individuals in the PDIWT group were analyzed. The majority of the individuals analyzed in both groups were women (56.3% in NCD group and 57.2% in PDIWT group; Table 2). In the NCD group, a higher proportion of individuals were 50–59 years old, whereas in the PDIWT group, individuals were equally distributed in the 40–49-years (29.2%) and 60–69-years (29.6%) age groups. The blood glucose test confirmatory demonstrated levels of likely or very likely DM in 57.3% of NCD; on the other hand, 72.0% of PDWIT had a borderline blood glucose result. Most individuals in both groups had a health behavior score <4 (81.3% and 79.0% for new cases and previously diabetic, resp.; Table 2). 

Among the NCD groups, 92.2% individuals referred utilization of health care services, significantly higher proportion than among the PDWIT group (33.1%; *P* < 0.001).  Table 3 details service utilization and pharmacological treatment in the two groups. In the group of NCD, blood glucose levels were associated with pharmacological treatment for diabetes (*P* < 0.001). In the PDWIT group, the outcomes of health care services utilization and pharmacological treatment were associated with the health behavior score (*P* < 0.05) and blood glucose levels in the screening program (*P* < 0.001).

When variables significantly associated to the evaluated outcomes in bivariate analysis (*P* < 0.05) were entered into a logistic regression model, and considering the NDC group as reference, the only factor significantly associated to health care services utilization was diabetes status (OR = 0.06; 95% CI: 0.03–0.13; Table 4). 

Factors significantly associated to pharmacological treatment were diabetes status (OR = 0.23; 95% CI: 0.14–0.37), healthy behavior score of 4 or higher (OR = 0.53; 95% CI: 0.34–0.83), altered blood glucose level (OR = 5.01; 95% CI: 2.38–10.57), and blood glucose in the level of likely and very likely diabetes (OR = 11.22; 95% CI: 6.85–18.40; Table 4).

## 4. Discussion

The Brazilian Ministry of Health implemented the National Program of Detection of Diabetes Mellitus as a component of the Plan for the Care of Diabetes and Hypertension, recognizing that the early identification, assistance, follow up of people with diabetes, and health care service utilization in the basic health units are crucial elements to health control. It was also recognized that assistance was not systematic in the country [5, 7, 8, 11]. 

The participation of previously diabetic individuals in the screening program showed the need for a better understanding of the components involved and possible influence in health service utilization. A possible explanation to justify this may be related to difficulties in the management of the disease and its treatment [13–15], or problems in the understanding of the disease itself [15], considering the low schooling of the population. The possibility that these individuals had taken part as a way to control glucose levels must be considered as well as occasional flaws in fulfilling the form. 

In both groups, females, individuals with incomplete elementary schooling, and those of 50–69 years of age predominated. The age of individuals that were not under treatment was similar to those considered new cases. For variables such as gender and age, results are in accordance with the observations of other studies [2, 3].

Most of the individuals presented less than four healthy behaviors (81.3% and 79.0%, NCD and PDWIT, resp.) demonstrating difficulties to establish and to keep non-pharmacological procedures. 

The analysis of demographic variables in the process of adhesion and health service utilization does not show results that may point out a specific profile of individuals. According to results of bivariate analysis, the only variable that reached statistical significance was schooling, considering pharmacological treatment as the outcome. However, health system utilization in this study, as in others [10, 16], was not influenced by age, gender, and schooling.

Health care service utilization was significantly higher among NCD, showing that the objective diagnosis and service utilization is achieved. If health care utilization was lower among PDIWT, special strategies should be necessary in this group in order to include them in the health system. The healthy behavior score and blood glucose levels in the screening were also associate to health service utilization in the bivariate analysis; however the association disappeared after logistic regression.

According to multivariate analysis, PDIWT group presents 23% of the chance of an NCD to treat the condition and has a healthy behavior score <4 corresponding to a 53% chance of pharmacologically treating the condition compared to individuals with four or more healthy behaviors. These data indicate a strong relationship between the acquisition and maintenance of healthy behaviors and the treatment of diabetes. The pharmacological treatment reaches a higher proportion of individuals with higher blood glucose levels considering that these individuals tend to be more symptomatic and, as a consequence, look for treatment. It is, however, interesting to emphasize that, when health service utilization is taken into consideration, glucose levels and healthy behavior scores were not statistically significant. 

Population-based studies on adhesion to treatment and health service utilization are not explored in the literature, making the importance of results of the program more evident. In the present study, the variable actually associated with health service utilization was the diagnosis of a new case. 

Possible limitations of this study must be considered. Information was obtained in a self-referred process by patients. Data were not directly obtained, nor a measurement was done or other registers were simultaneously employed. Other authors underscored this kind of limitation [17]. We should also consider the possibility of some inadequacy in the answers on the form to select individuals with diabetes, causing problems in further orientations to this group. The omission of this information may have occurred in order to obtain a free glucose test. Another important point to be considered is that, as mentioned by other authors [9], in general individuals that do not adhere to components of treatment tend to be more sincere. The period of time between the program and the active surveillance (15 to 19 months) may have caused differences in the declarations. There is the possibility of memory bias, since sick individuals tend to better recall their behavior.

In summary, results obtained are in accordance with those of the literature, showing that demographic variables do not make a specific profile of adhesion to pharmacological treatment and health service utilization. The greater service utilization verified among NCD indicates a relevant result of the National Plan and, particularly, of the Program, as a screening strategy and further service utilization. Part of the PDIWT group was recovered by the health system; however, strategies specially directed to this group deserve consideration. Importantly, the work developed in health system by the available programs should be revisited and optimized in order to obtain a better support for patients with chronic diseases such as, according to this study, diabetes.

## Figures and Tables

**Table 1:** Classification of screening test results and recommendations made to individuals who participated in the BNPSPD, brazil, 2001.

	mg/dL	Categories	Recommendation
Fasting capillary glucose	<100	Normal	Repeat test in 3 years
	100 to 125	Borderline	Schedule future appointment
	126 to 199	Altered	Order fasting serum glucose and recommend return medical appointment
	≥200	Likely DM	Order fasting serum glucose and schedule future appointment
	≥270	Very likely DM	Immediate consultation with physician

Nonfasting capillary glucose	<140	Normal	Repeat test in 3 years
	140 to 199	Borderline	Schedule future appointment
	≥200	Likely DM	Order fasting serum glucose and recommend return medical appointment
	≥270	Very likely DM	Immediate consultation with physician

BNPSPD: Brazilian Nationwide Population Screening Program for Diabetes.

**Table 2:** Clinical and demographic characteristics of BNPSPD participants considering the two groups analyzed. Brazil, 2001.

	New cases (NCD)	Previously diabetic individuals without treatment (PDWIT)
	*N* = 497	*N* = 257
	(%)	(%)
*Gender*		
Male	217 (43.7)	110 (42.8)
Female	280 (56.3)	147 (57.2)
*Age (years)*		
40–49	127 (25.6)	75 (29.2)
50–59	160 (32.2)	69 (26.8)
60–69	131 (26.4)	76 (29.6)
70 or more	79 (15.9)	37 (14.4)
*Schooling*		
Illiterate	121 (24.6)	55 (21.5)
Elementary education incomplete	282 (57.4)	139 (54.3)
Elementary education complete or +	88 (17.9)	62 (24.2)
*Region*		
North	22 (4.4)	17 (6.6)
Northeastern	138 (27.8)	44 (17.1)
Midwestern	28 (5.6)	24 (9.3)
Southeastern	236 (47.5)	119 (46.3)
South	73 (14.7)	53 (20.6)
*Glucose level * *		
Borderline	137 (27.6)	185 (72.0)
Altered	75 (15.1)	18 (7.0)
Likely and very likely	285 (57.3)	54 (21.0)
*Healthy score*		
<4	404 (81.3)	203 (79.0)
≥4	93 (18.7)	54 (21.0)

*Borderline ≥ 100 < 126 mg/dL, altered ≥ 126 < 200 mg/dL, likely and very likely ≥200 mg/dL.

BNPSPD: Brazilian Nationwide Population Screening Program for Diabetes.

**Table 3:** Comparison of factors potentially associated to healthcare service utilization and pharmacological treatment in the two groups analyzed. Brazil, 2001.

	New cases (NCD)		Previously diabetic individuals without treatment (PDWIT)	
	Service utilization	Pharmacological treatment	Service utilization	Pharmacological treatment
*Gender*				
Male	197/217 (90.8)	168/217 (77.4)	41/110 (37.3)	39/110 (35.5)
Female	261/280 (93.2)	221/280 (78.9)	44/147 (29.9)	43/147 (29.3)
*Age (years)*				
40–49	113/127 (89.0)	92/127 (72.4)	21/75 (28.0)	19/75 (25.3)
50–59	152/160 (95.0)	126/160 (78.7)	23/69 (33.3)	22/69 (31.9)
60–69	122/131 (93.1)	104/131 (79.4)	29/76 (38.2)	28/76 (36.8)
70 or more	71/79 (89.9)	67/79 (84.8)	12/37 (32.4)	13/37 (35.1)
*Schooling*				
Illiterate	107/121 (88.4)	94/121 (77.7)	14/55 (25.5)	15/55 (27.3)
Elementary education incomplete	266/282 (94.3)	229/282 (81.2)	48/139 (34.5)	48/139 (34.5)
Elementary education completed or +	79/88 (89.8)	60/88 (68.2)	23/62 (37.1)	19/62 (30.6)
*Healthy behaviour*				
Score <4	376/407 (93.1)	323/407 (80.0)	76/203 (37.4)^ +^	73/203 (36.0)^ +^
Score ≥4	82/93 (88.2)	66/93 (71.0)	9/54 (16.7)^ +^	9/54 (16.7)^ +^
Glucose level**				
Borderline	125/137 (91.2)	78/137 (56.9)*	25/185 (13.5)*	22/185 (11.9)*
Altered	68/75 (90.7)	57/75 (76.0)*	17/18 (94.4)*	15/18 (83.3)*
Likely and very likely DM	265/285 (93.0)	254/285 (89.1)*	43/54 (79.6)*	45/54 (83.3)*
*Region*				
North	19/22 (86.4)	13/22 (59.1)	9/17 (52.9)	8/17 (47.1)
Northeastern	118/138 (85.5)	103/138 (74.6)	11/44 (25.0)	12/44 (27.3)
Midwestern	27/28 (96.4)	24/28 (85.7)	8/24 (33.3)	9/24 (37.5)
Southeastern	226/236 (95.8)	193/236 (81.8)	39/119 (32.8)	38/119 (31.9)
South	68/73 (93.2)	56/73 (76.7)	18/53 (34.0)	15/53 (28.3)

^+^ *P* < 0,05.

*P < 0,001.

**Borderline ≥ 100 < 126 mg/dL, altered ≥ 126 < 200 mg/dL likely and very likely ≥200 mg/dL.

**Table 4:** Factors associated to healthcare service utilization and pharmacological treatment in multivariate analysis. Brazil, 2001.

	Service utilization OR (95% CI)	Pharmacological treatment OR (95% CI)
*Diabetes status*		
New cases	1	1
Previously diabetic individuals under treatment	0.06 (0.03–0.13)	0.23 (0.14−0.37)
*Healthy behavior score *		
≥4	1	1
<4	0.62 (0.34–1.13)	0.53 (0.34–0.83)
*Glucose level at the screening * *		
Borderline	1	1
Altered	2.22 (0.62–7.93)	5.01 (2.38−10.6)
Likely and very likely DM	1.20 (0.59–2.46)	11.2 (6.85−18.4)

*Borderline ≥ 100 < 126 mg/dL, altered ≥ 126 < 200 mg/dL, likely and very likely ≥200 mg/dL.

## References

[1] Shaw JE, Sicree RA, Zimmet PZ (2010). Global estimates of the prevalence of diabetes for 2010 and 2030. *Diabetes Research and Clinical Practice*.

[2] International Diabetes Federation (2009). *IDF Diabetes Atlas*.

[3] King H, Aubert RE, Herman WH (1998). Global burden of diabetes, 1995–2025: prevalence, numerical estimates, and projections. *Diabetes Care*.

[4] Wild S, Roglic G, Green A, Sicree R, King H (2004). Global prevalence of diabetes: estimates for the year 2000 and projections for 2030. *Diabetes Care*.

[5] Barbosa RB, Barcelo A, Machado CA (2001). National campaign to detect suspected diabetes cases in Brazil: a preliminary report. *Revista Panamericana de Salud Publica*.

[6] Nucci LB, Toscano CM, Maia AL (2004). A nationwide population screening program for diabetes in Brazil. *Revista Panamericana de Salud Publica*.

[7] (2001). *Plano de Reorganização da Atenção à Hipertensão Arterial e ao Diabetes Mellitus: Hipertensão Arterial e Diabetes Mellitus*.

[8] Toscano CM, Duncan BB, Mengue SS (2008). Initial impact and cost of a nationwide population screening campaign for diabetes in Brazil: a follow up study. *BMC Health Services Research*.

[9] Sabaté E (2003). *Adherence to Long-Term Therapies: Evidence for Action*.

[10] Di Matteo MR (2004). Variations in patients’ adherence to medical recommendations: a quantitative review of 50 years of research. *Medical Care*.

[11] (2004). *Avaliação do Plano de Reorganização da Atenção à Hipertensão e ao Diabetes Mellitus no Brasil*.

[12] Di Matteo MR, Haskard KB, Williams SL (2007). Health beliefs, disease severity, and patient adherence: a meta-analysis. *Medical Care*.

[13] Engelgau MM, Narayan KM, Saaddine JB, Vinicor F (2003). Addressing the burden of diabetes in the 21st century: better care and primary prevention. *Journal of the American Society of Nephrology*.

[14] Narayan KM, Benjamin E, Gregg EW, Norris SL, Engelgau MM (2004). Diabetes translation research: where are we and where do we want to be?. *Annals of Internal Medicine*.

[15] Nam S, Chesl C, Stotts NA, Kroon L, Janson LS (2011). Barriers to diabetes management: patient and provider factors. *Diabetes Research Clinical Practice*.

[16] Eakin EG, Bull SS, Glasgow RE, Mason M (2002). Reaching those most in need: a review of diabetes self-management interventions in disadvantaged populations. *Diabetes/Metabolism Research and Reviews*.

[17] Satterfield DW, Volansky M, Caspersen CJ (2003). Community-based lifestyle interventions to prevent type 2 diabetes. *Diabetes Care*.

